# Potential health benefits of insect bioactive metabolites and consumer attitudes towards edible insects

**DOI:** 10.1038/s41538-025-00549-x

**Published:** 2025-09-26

**Authors:** Jackson Ishara, Idaresit Ekaette, Rehema Matendo, Grant Vandenberg, Saliou Niassy, Karume Katcho, John Kinyuru

**Affiliations:** 1 Department of Food Science and Technology, Université Evangélique en Afrique, P.O. Box 3323, Bukavu, Democratic Republic of the Congo; 2 Department of Food Science and Technology, Jomo Kenyatta University of Agriculture and Technology, P.O. Box 62000-00200, Nairobi, Kenya; 3 Faculty of Agriculture and Environmental Sciences, Université de Kaziba, P.O. Box 2106, Bukavu, Democratic Republic of the Congo; 4 Department of Food Science and Agricultural Chemistry, McGill University, Ste-Anne-de-Bellevue, Quebec, H9X 3V9 Canada; 5 Department of Bioresource Engineering, McGill University, Ste-Anne-de-Bellevue, Quebec, H9X 3V9 Canada; 6 Department of Environmental and Agronomic Sciences, Université Officielle de Bukavu, P.O. Box 570, Bukavu, Democratic Republic of the Congo; 7 Chair of Educational Leadership (CLE) in Primary Production and Processing of Edible Insects (CLEIC), Quebec, Canada; 8 Département des sciences animales, Faculté des sciences de l’agriculture et de l’alimentation, Université Laval, 2425 rue de l’Agriculture, Québec, QC G1V 0A6 Canada; 9 Inter-African Phytosanitary Council of African Union (AU-IAPSC), P.O Box 4170, Yaoundé, Cameroon; 10 Department of Zoology and Entomology, Faculty of Natural and Agricultural Sciences University of Pretoria, Hatfield, Pretoria, Gauteng South Africa; 11 Faculty of Agriculture and Environmental Sciences, Université Evangélique en Afrique, Bukavu, 3323 Democratic Republic of the Congo; 12 Centre de Recherche en Géothermie, Bukavu, 327 Democratic Republic of the Congo; 13 African Institute for Capacity Development (AICAD), P.O. Box 46179-00100, Nairobi, Kenya

**Keywords:** Biochemistry, Biotechnology

## Abstract

Particular attention has been paid to the nutritional potential of edible insects as well as the health benefits associated with their bioactive compounds. This paper focused on an in-depth review compiling the most recent information on health benefits of insect bioactive metabolites as well as their purification and identification, in addition to consumer attitudes towards edible insects. It was found that, insect bioactive metabolites, including marcocarpal, grandinol, trolline, pancratistatin, narciclasin, ungeremin, cantharidin, cordycepin, roseoflavin, lecithin, reblastatin, chitin, chitosan and desmosterol deemed to have biological activities, such as tumor suppression, anticancer, antihypertensive, anti-inflammatory, antioxidant, immunomodulator, neuroprotective, glycemic and lipid regulation, blood pressure reduction, regulation of intestinal bacterial flora and cardiovascular protection among others. Furthermore, proper sample preparation and extraction is the first step in the purification of bioactive metabolites from edible insects. After concentration, bioactive metabolites are purified using chromatographic and separation techniques including High-Performance Liquid Chromatography (HPLC), Gas Chromatography (GC), Thin-Layer Chromatography (TLC), Size-Exclusion Chromatography (SEC). Finally, their nutritional potential, health benefits, environmentally friendly, great taste, traditions, taboo, safety concerns, unpleasant past experiences, allergies, and unnaturalness are among the main factors influencing attitudes towards insect consumption.

## Introduction

With a rapidly growing world population^1^ and the goal of promoting healthier as well as sustainable food systems^2^, there is a growing demand for alternative proteins^3^. This situation is exacerbated by the scarcity of essential arable land^4^, environmental pressures linked to the uncertainties of climate change^5,6^. Edible insects are thus seen as a formidable alternative to address the issues of global food insecurity^7^ for their nutritional potential^8,9^, taste^10^, economic benefits^11,12^, environmental benefits^13^, as well as their potential health benefits^14^.

In many parts of the world, entomotherapy is used as medicine and is an important alternative to modern therapy through their bioactive metabolites including pancratistatin, narciclasin, ungeremin, cantharidin, cordycepin, roseoflavin, lecithin, reblastatin, chitin, and chitosan^15,16^. These bioactive compounds present important physiological effects on living organisms through their physiological properties encompassing anti-obesity, antihypertensive, antithrombotic, antioxidant, hypocholesterolemic, antimicrobial, opioid, cytomodulatory, anti-inflammatory, cardioprotective, immunomodulatory, antiangiogenic, and immunomodulatory activities^14,17^.

Given their diverse functions, high bioavailability and efficacy even at low concentrations, bioactive compounds attract a great deal of attention, although some bioactive compounds are naturally present in isolation, many are hidden within the intact structure^18^. Even though effort is being made, consumer attitudes and willingness to consume insects remain a major challenge in many societies^19^, due to traditions, superstitions and taboos as well as familiarity with insect^20^, their appearance and great taste^8,21^.

Considering the attention paid to insects as food and feed, this review compiled the most recent information focusing on health benefits of insect bioactive metabolites as well as their purification and identification, and finally a particular attention was paid to sensory attributes and consumer attitudes towards edible insects.

## Potential health benefits of insect bioactive metabolites

**Fig. 1:**
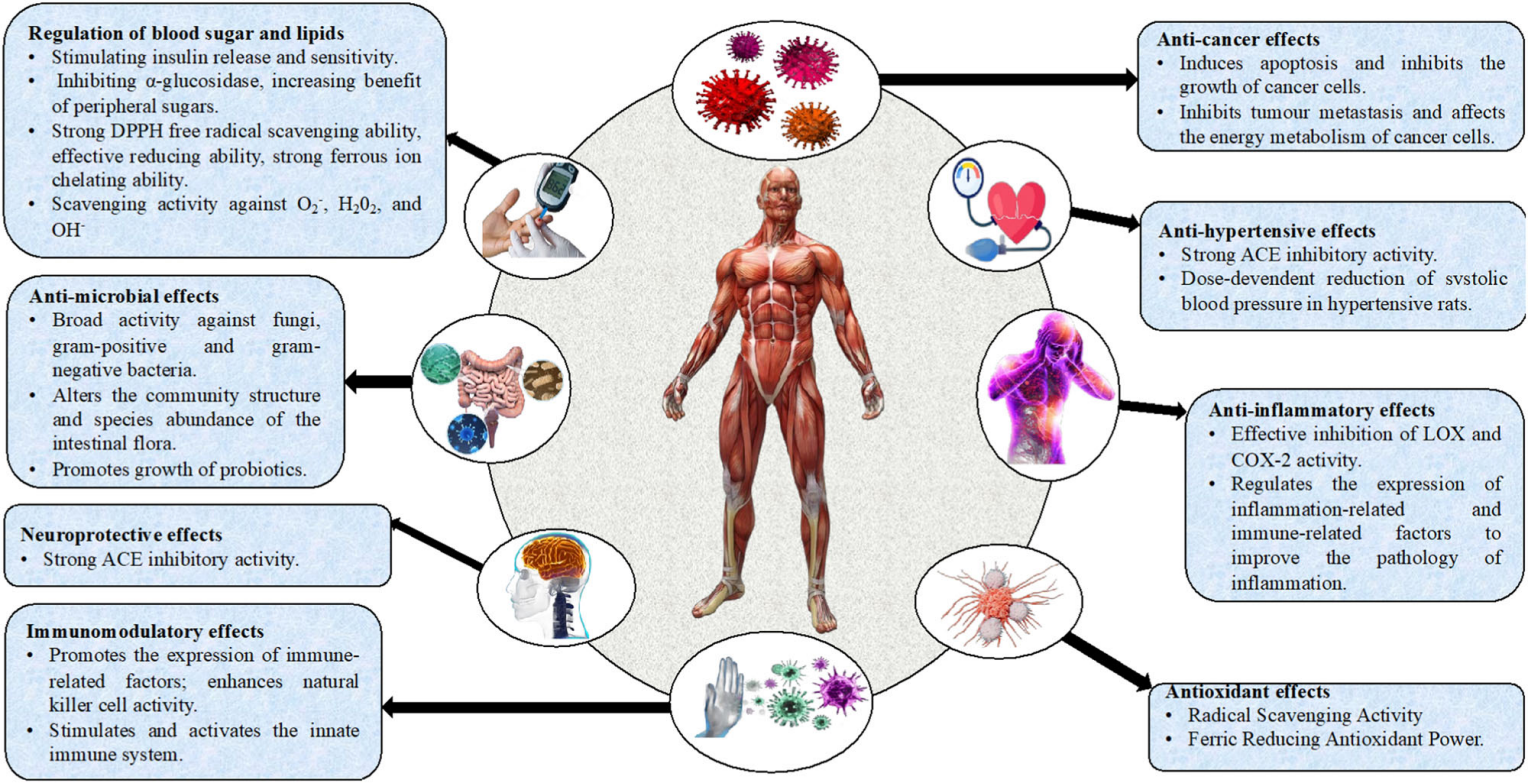
Health benefits of insect bioactive metabolites and their potential mechanisms.

**Fig. 2:**
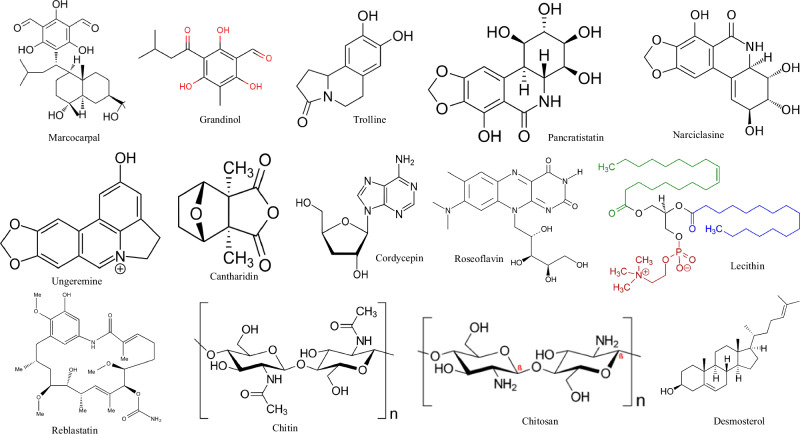
Chemical structures of selected bioactive metabolites found in edible insects.

Insects are characterized by several bioactive metabolites, including marcocarpal, grandinol, trolline, pancratistatin, narciclasine, ungeremine, cantharidin, cordycepin, roseoflavin, lecithin, reblastatin, chitin, chitosan and desmosterol (Fig. 1), which confer a variety of beneficial biological activities to human health, including tumor suppression, anti-cancer, anti-hypertensive, anti-inflammatory, antioxidant, immunomodulatory, neuroprotective, blood sugar and lipid regulation, blood pressure reduction, regulation of intestinal bacterial flora and cardiovascular protection (Table 1). While Fig. 2 illustrates the biological activities of bioactive insect metabolites and their mechanisms of activity, detailed information associating insect species to their bioactive metabolites is depicted in Table 1.

**Table 1:** Potential health benefits of bioactive metabolites found in edible insects

Scientific name	Metabolite	Biological activity and study description	Reference
**Blattodea**			
*Macrotermes natalensis*	Actinomycin D	Antibacterial, antitumor (In vitro). Assessment using two different bioassays.	Benndorf et al.^22^
	Rubterolone A–F	Farnesyl-protein transferase inhibitor (In vitro). Assessment using two different bioassays.	Benndorf et al.^22^
	Barceloneic acid A		Benndorf et al.^22^
*Periplaneta americana*	Periplatins A–D	Antiproliferative (In vitro). Significant cytotoxic activities in HepG2 and MCF-7 cells with IC_50_ values in the ranges 6.41-23.91 μM and 6.67-39.07 μM was observed. Isolated from the 70% ethanol extract of the whole body.	Luo et al.^23^
*Polyphaga plancyi*	Eupolyphagin	Antiproliferative. Two series of novel 2-substituted-4-amino-6-halogenquinolines 8a-l and 13a-H were designed, synthesized and evaluated for their antiproliferative activity against H-460, HT-29, HepG2 and SGC-7901 cancer cell lines in vitro. IC_50_ values of 0.03 μM, 0.55 μM, 0.33 μM and 1.24 μM.	Jiang et al.^112^
*Polyphaga plancyi*	Plancyamides A; and B, plancypyrazine A; plancyols A and B	Antiproliferative activity evaluated toward extracellular matrix in animal model (rat renal proximal tubular cells), human cancer cells (K562, A549, and Huh7), EV71, ROCK2, JAK3, DDR1, and coagulation.	Zhu et al.^113^
*Odontotermes formosanus*	5-Hydroxyramulosin and biatriosporin M	Antifungal and antibacterial. The phylogenetic diversity of fungi isolated from *O. formosanus* was investigated by dilution-plate method, combined with morphological characteristics and 5.8S rDNA sequencing. The antimicrobial activities of all endophytic fungi extracts were tested by using the filter paper method against *E. coli* (ATCC 8739), *B. subtilis* (ATCC 6633), *S. aureus* (ATCC 6538), and *C. albicans* (ATCC 10231). Medium inhibitory activities against *B. subtilis* and *S. aureus*, with the IZD range of 8.32-9.13 mm was observed.	Xu et al.^64^
	1-(2,5-Dihydroxyphenyl)-3-hydroxybutan- 1-one		Xu et al.^64^
	Roseoflavin		Xu et al.^64^
	Roseoflavin		Zhou et al.^65^
	8-methylamino-8-demethyl-d-riboflavin		Zhou et al.^65^
*Periplaneta americana*	Isocoumarins periplatins A–D	Cytotoxic activities against human liver (HepG2) and breast cancer (MCF-7) cells with IC_50_ values in the ranges 6.41-23.91 μM and 6.67-39.07 μM.	Luo et al.^23^
*Polyphaga plancyi*	Plancyamide A; Plancypyrazine A; Plancypyrazine B; Plancyol A	Antiproliferative activity evaluated toward extracellular matrix in rat renal proximal tubular cells, human cancer cells (K562, A549, and Huh7), EV71, ROCK2, JAK3, DDR1, and coagulation.	Zhu et al.^113^
*Macrotermes natalensis*	Natalamycin A	Bioassay-guided fractionation based on antifungal activity led to the isolation of natalamycin A, Geldanamycin and Reblastatin (In vitro).	Kim et al.^114^
	Geldanamycin		
	Reblastatin		
	Termisoflavones A–C	Improved cisplatin-induced kidney cell damage to 80% of the control value at a cisplatin dose of 25 μM (In vitro).	Kang et al.^115^
	Isoflavanoids		
	Dentigerumycins B–D	Cisplatin-induced cytotoxity. The structures of the complex nonribosomal peptide synthetase-polyketide synthase (NRPS/PKS) hybrid bioactive compounds were determined by 1D- and 2D-NMR spectroscopy, high-resolution mass spectrometry, and circular dichroism (CD) spectroscopy.	Wyche et al.^116^
	Macrotermycin A–C	Bioassay-guided metabolomic analyses. Macrotermycins A and C had antibacterial activity against human-pathogenic *S. aureus* and a selective antifungal activity against a fungal parasite of the termite fungal garden.	Beemelmanns et al.^31^
	Banegasine, Cyclo-NMe-L-3,5-dichlorotyrosine-Dhb	Antifungal (In vitro). Antifungal activity assessed using two different bioassays.	Benndorf et al.^22^
	Rubrominin A–B		
	Microtermolide A–B	Farnesyl-protein transferase inhibitor. Microtermolides A and B were isolated from a *Streptomyces sp*. strain associated with fungus-growing termites. 1D- and 2D-NMR spectroscopy and high-resolution mass spectrometry were used to determine the structures of A and B.	Carr et al.^117^
	Drimenol-type sesquiterpenes	Antibacterial (In vitro). Formation of two structurally related monocyclic sesquiterpenes (nectrianolines) was catalyzed by heterologously expressed enzymes potentially involved in terpene biosynthesis.	Kreuzenbeck et al.^63^
	Natalenamides A–C	Inhibitory effects on 3-isobutyl-1-methylxanthine (IBMX)-induced melanin production (In vitro).	Lee et al.^32^
*Macrotermes spp*.	Efomycin K, Efomycin L, Efomycin M, Efomycin G and Elaiophylin	Antifungal. Phylogenetic analysis of gene cluster domains was used to provide a biosynthetic rational for these new derivatives.	Klassen et al.^72^
	Efomycin M	Inhibited selectin-mediated leukocyte rolling In vivo inflammatory skin models using transplanted human skin biopsies.	von Bonin et al.^118^
	Roseoflavin, 8-methylamino-8-demethyl-; D-riboflavin; Natalamycin; Termisoflavones A-C	Antibacterial and antifungal (In vitro).	Zhou et al.^65^
**Coleoptera**			
*Blaps japanensis*	Blapsols A–D	Structures determined by means of spectroscopic and X-ray crystallographic methods. Chiral HPLC was used to separate (-)- and (+)-enantiomers of compounds 1-4, which were isolated from *Blaps japanensis* as racemic mixtures. Effects towards COX-2 with IC_50_ values in the range of 1.3-17.8 μM were observed. Multiple assays including anti-tumor, anti-inflammatory, and renal protection activities were determined using in vitro biological evaluations.	Seabrooks & Hu^17^
			Yan et al.^42^
*Catharsius molossus*	Molossusamides A–C	Antibacterial and anti-inflammatory (In vitro). Cytotoxicity, MDCK cell based anti-influenza, EV71 inhibition and cyclooxygenase inhibitory assays were used to evaluate the biological activities of all the compounds.	Lu et al.^67^
*Cantharis vesicatoria*	Cantharidin and norcantharidin	Caprine luteal cell steroidogenesis inhibitor. Steroidogenic effects of cantharidin and norcantharidin (0.1, 1.0, and 10 μg ml^-1^) were assessed from luteal cells isolated from corpora lutea of native Taiwan goats maintained in vitro and treated for 4 and 24 h.	Twu et al.^119^
*Copris tripartitus*	Tripartin	Histone H3 lysine 9 demethylase KDM4 inhibitor (In vitro).	Kim et al.^120^
	Coprisamides A–B	Quinone reductase inducer (In vitro).	Kim et al.^121^; Um et al.^122^
	Coprisidin A	Na + /K+ ATPase inhibitor (In vitro).	
	Coprisidin B	NAD(P)H:quinone oxidoreductase 1 inducer (In vitro).	
	Tripartilactam	Its structure was elucidated by the combination of NMR, MS, UV, and IR spectroscopy and multistep chemical derivatization. Tripartilactam was evaluated as a Na + /K+ ATPase inhibitor (IC_50_ = 16.6 μg/mL) in vitro.	Park et al.^123^
*Dendroctonus frontalis*	Mycangimycin, Frontalamide A, and Frontalamide B	Antimalarial (In vitro). Genome analyses and genetic manipulation of the producing organism led to the identification of the frontalamide biosynthetic gene cluster and several biosynthetic intermediates.	Blodgett et al.^124^
*Holotrichia diomphalia*	Tricin, palmitinic acid; eicosane	Antifungal (In vitro). Chemical compositions of the fatty oils were obtained by two different methods and determined by GC/MS.	Dong et al.^73^
*Hycleus lunata*	Cantharidin	Antitumor in mice model. Cantharidin treatment induced abnormal mitochondrial characteristics, with a decrease in mitochondrial glutathione, succinate dehydrogenase activity, mitochondrial membrane potential, and induced apoptosis and necrosis in DL cells.	Prasad & Verma^125^
	Cantharidin and norcantharidin	SoNar, a highly responsive NAD + /NADH sensor, allows high-throughput Metabolic screening of anti-tumor agents in vitro and in vivo.	Zhao et al.^126^
	Palasonin and Palasoninimide	Whole specimens of *Hycleus lunata* or body components were hydrolysed with 50-300 µl 6 N hydrochloric acid at 120 °C for 4 h. Each *Hycleus* extract was injected in a capillary glass chromatograph. Protein phosphatase 2 A inhibitors.	Dettner et al.^127^
	Cantharidin	Antiproliferative; immunomodulatory	Lang & Lang^128^
*Mylabris phalerata*	Cantharidin and norcantharidin	Caprine luteal cell steroidogenesis inhibitor. Steroidogenic effects of cantharidin and norcantharidin (0.1, 1.0, and 10 μg ml^-1^) were assessed from luteal cells isolated from corpora lutea of native Taiwan goats maintained in vitro and treated for 4 and 24 h.	Twu et al.^119^
*Onthophagus lenzii*	Lenzimycins A–B	Selective isolation of bacterial strains associated with the dung beetle, *O. lenzii*. PTH23 and lenzimycins A and B (1-2) inhibited the growth of *Bacillus sp*. CCARM 9248 and of some human pathogenic bacteria, including *E. faecium* and certain strains of *E. faecalis*.	An et al.^68^
*Tenebrio molitor*	Defatted larvae	Diet enriched with defatted larvae of the mealworm *Tenebrio molitor* (TM) endowed with ACE inhibitory activity was studied in both spontaneously hypertensive rats (SHR) and in the age-matched normotensive Wistar Kyoto strain fed for 4 weeks with standard laboratory rodent chow supplemented with or without TM or captopril. In SHR, the TM diet led to a significant reduction in blood pressure, heart rate and coronary perfusion pressure, as well as an increase in red blood cell glutathione/glutathione disulphide ratio. Rat brain slices of SHR were more resistant to oxidative stress with lower levels of inflammatory cytokines.	Pessina et al.^30^
	Peptides CSR, APVAH, PAALST, AAGAPP AR and APYF	Fractionation and identification of dipeptidyl peptidase IV (DPP-IV) and α-glucosidase inhibitory peptides was carried out. Peptides from 500 to 1600 Da showed the highest level of DPP-IV inhibition with IC_50_ value of 0.91 mg ml^−1^ and peptides below 500 Da showed the highest level of α-glucosidase inhibition with IC_50_ value of 2.58 mg ml^−1^.	Rivero-Pino et al.^129^
*Tenebrio molitor* Larvae	Peptides NYVADGLG, AAAPVAVAK, YDDGSYKPH	Antioxidant and anti-inflammatory activities of peptide fractions from hydrolysates obtained by in vitro gastrointestinal digestion. Peptide fraction from the *T. molitor* protein preparation revealed the highest Fe2+ chelating ability with EC_50_ value 2.21 μg mL^−1^ and the highest reducing power (0.198). ACE, pancreatic lipase, and α-glucosidase inhibitory in vitro were reported.	Zielinska et al.^130,131^
**Diptera**			
*Hermetia illucens*	Pyrone derivatives	Compounds from *C. multifidum*, a fungus with moderate antimicrobial activity isolated from *H. illucens* gut microbiota. Extract from *C. multifidum* resulted in a moderate activity against a strain of methicillin-resistant *S. aureus* (MRSA). Bioguided isolation of the extract showed the characterization of six α-pyrone derivatives (1-6) and one diketopiperazine (7). Among these compounds, 5,6-dihydro-4-methoxy-6-(1-oxopentyl)-2H-pyran-2-one (4) showed the greatest activity with IC_50_ = 11.4 ± 0.7 μg/mL and MIC = 62.5 μg/mL against MRSA.	Correa et al.^69^
	Diketopiperazine		
	Photoinduced, melanins and ommochromes	Antibacterial, antifungal, antioxidant, and anti-inflammatory. Isolated pigments were analyzed by HPLC and represented a mixture of several ommochromes of the ommatin series.	Dontsov et al.^44^; Richter et al.^34^
	Defensins-DLP2 and DLP4	Peptides-DLP2 and DLP4 showed potent antimicrobial activity against Gram-positive bacteria. The survival of mice challenged with MRSA were 80-100% at the doses of 3-7.5 mg/kg DLP2 or DLP4. The bacterial translocation burden over 95% in spleen and kidneys and serum pro-inflammatory cytokines levels were reduced by DLP2 and DLP4, and promoted anti-inflammatory cytokines levels; as well as improved lung and spleen injury.	Li et al.^47^
	Bioactive peptides	Antioxidant activity. Proteomics-based analysis was used to characterize and quantify functional proteins and bioactive peptides produced from the enzymatic digestion of *H. illucens* fed with food wastes. The 60S ribosomal protein L5 (RpL5) in BSF interacted with a variety of ribosomal proteins and played a key role in the glycolytic process (AT14039p). Higher antioxidant activity was observed in peptide sequences such as GYGFGGGAGCLSMDTGAHLNR, VVPSANRAMVGIVAGGGRIDKPILK, AGLQFPVGR, GFKDQIQDVFK, and GFKDQIQDVFK.	Lu et al.^43^
*Musca domestica*	Protein-enriched fraction (PEF)	Antiviral, immunomodulatory, and free radical scavenging activities of PEF isolated from the larvae of the housefly was assessed. Infection of avian influenza virus H9N2 and had a virucidal effect against the multicapsid nucleopolyhedrovirus of the alfalfa looper, *A. californica* Speyer inhibited in vitro. Excellent scavenging activity for 1,1-diphenyl-2-picrylhydrazyl and superoxide anion radicals were similar to those of ascorbic acid.	Ai et al.^48^
**Hemiptera**			
*Aspongopus chinensis*	(±)-Aspongamide A	Inhibition of Smad3 phosphorylation in transforming growth factor-β1 (TGF-β1) induced rat renal proximal tubular cells and suppressed extracellular matrix expression in mesangial cells under diabetic conditions.	Yan et al.^132^; Shi et al.^133^
	Aspongopusamides A–D	Anti-inflammatory and therapeutic agents against renal protection in high-glucose-induced mesangial cells and COX-2 inhibition.	Shi et al.^133^
*Kerria lacca*	Shellolic acid A	Isolated from Shellac. HR-ESI-MS, UV, IR, 1D and 2D NMR methods were used to elucidate the structures. The Mosher’s method, circular dichroism (CD) and optical rotation analyses were used for absolute configurations. Cytotoxic and anti-bacterial activities of the isolates were evaluated. Inhibitory activity against *B. subtilis* with the MIC value of 0.1 mg/mL.	Lu et al.^53^
**Hymenoptera**			
*Apis mellifera*	Lupeol, lupenone; Lupeol acetate	This paper proposed a new accelerated solvent extraction method to obtain low-polarity actives with a high cytotoxic profile from red propolis. To identify the chemical compounds, the extracts were analyzed by gas chromatography-mass spectrometry. To profile the cytotoxicity of the bioactives obtained, the 3-(4,5-dimethyl-2-thiazole)-2,5-diphenyl-2-H-tetrazolium bromide colorimetric assay was performed on different tumor cell lines (HCT116 and PC3). The extract obtained at 70 °C and a 10-minute extraction cycle showed the highest cytotoxic activity against the cell lines tested.	de Carvalho et al.^134^
*Apis mellifera* pupae	Polypeptide components (BPP-21 and BPP-22)	Immunomodulatory activity in vivo and in vitro. Protein hydrolysis using alkaline protease yielded novel bee chrysalis polypeptides (BPP). A diethylaminoethylsepharose fast-flow column and a Sephadex G-25 column were used to isolate and purify two purified polypeptide components (BPP-21 and BPP-22). Identification was made using HPLC size exclusion and amino acid composition analyses. BPP-22 significantly increased the delayed-type hypersensitivity reaction, cytokine levels (interleukin (IL)-2 and interferon (IFN)-γ), immunoglobulin (Ig) levels (IgA, IgG and IgM) and common blood indices in immunosuppressed mice treated with cyclophosphamide. BPP-22 potentially promoted cytokine secretion (IL-2, tumor necrosis factor-α and IFN-γ) and nitric oxide production by increasing homologous mRNA expression, and could exert immunomodulatory activity by increasing phosphorylation of ERK and p38, and modulating expression of intranuclear transcription factors (EIK-1, MEF-2 and CREB) in the MAPK signaling pathway.	Chen et al.^50^
*Polyrhachis dives*	(±)-Polyrhadopamine A, ( ± )-Polyrhadopamine B, ( ± )-Polyrhadopamine C, trolline, ( ± )-Polyrhadopamine C; β-carboline-3-carboxamide 5-(3-indolylmethyl)- nicotinsaureamide	Thirteen non-peptidic nitrogen compounds were isolated from *Polyrhachis dives*. Multiple assays, including renal protection, T and B cell proliferation, TNF-α, COX-1, COX-2 and Jak3 kinase inhibition were used to determine their biological potential. Several of these non-peptidic nitrogen compounds have shown immunosuppressive, anti-inflammatory and renoprotective properties.	Tang et al.^35,132^
*Polybia paulista*	Polybioside	From the venom of the social wasp *P. paulista* was used to isolate the polybioside (1) with a structure attributed to 3,4,5-trihydroxy-6-(hydroxymethyl) tetrahydro-2H-pyran-2-yl 3-(1H-imidazol-4-yl) propanimidate by NMR and MS protocols. Application of the polybioside in the rat brain, followed by detection of c-Fos protein expression in certain brain regions, indicated that the compound is neuroactive in a number of brain areas, and induces convulsions in rats, even when applied peripherally.	Saidemberg et al.^51^
*Tetraponera rufonigra*	Tetraponerins	A general stereocontrolled methodology was applied to access all natural tetraponerins from (+)-T1 to (+)-T8. Their anticancer activity against four different human cell lines, notably the MCF-7 breast carcinoma cell line, was observed through the cytotoxic activity of (+)-T7.	Bosque et al.^52^
*Solenopsis invicta*	Solenopsin A	The SVR angiogenesis assay was used to isolate solenopsine, an alkaloid component of the fire ant (*Solenopsis invicta*), which inhibited phosphatidylinositol-3-kinase signaling and angiogenesis.	Arbiser et al.^135^
**Lepidoptera**			
*Bombyx mori*	Hexapeptide	Ultramicroprocessed silkworm chrysalis protein alkalase hydrolysate was used to purify a novel immunomodulatory hexapeptide using sephadex gel filtration chromatography and reverse-phase high-performance liquid chromatography. The purified peptide had a molecular mass of 656.17 Da and an amino acid sequence of Pro-Asn-Pro-Asn-Thr-Asn (PNPNTN). Splenocyte proliferation was 87.35% in the presence of 100 μg/ml of purified peptide.	Li et al.^49^
*Bombyx mori* pupa	Peptides SQSPA, QPGR, NSPR, QPPT, KHV and GNPWM.	α-glucosidase in silico SGID, simulated gastrointestinal digestion (pepsin, trypsin, pancreatin, depending on the authors).	Zhang et al.^136^
*Bombyx mori*	Alkaloids and flavonoids	The anti-diabetic effects of the α-glucosidase inhibitor 1-deoxynojirimycin (DNJ) isolated from *B. mori* were evaluated in Otsuka Long-Evans Tokushima Fatty (OLETF) rats, an established animal model of human type 2 diabetes mellitus, and Long-Evans Tokushima Otsuka (LETO) rats were used as control. DNJ treatment resulted into significant anti-diabetic effects in LETO rats, with significant improvements in fasting blood glucose and glucose tolerance and, in particular, an increase in insulin sensitivity.	Kong et al.^137^; Liu et al.^138^
*Byasa polyeuctes*	Papilistatin	A novel inhibitor of cancer cell growth, papilistatin was isolated by bioassay-guided separation of an extract from the wings of a Taiwanese butterfly, *Byasa polyeuctes termessa*. Analysis of 1D and 2D NMR spectra and by HRMS was used to determine its structure. Papilistatin showed cancer cell growth inhibition with GI_50_’s of 0.093-3.5 μg/mL against a panel of six human and murine P388 cancer leukemia cell lines, as well as antibacterial activity.	Pettit et al.^71^
**Orthoptera**			
*Brachystola magna*	Pancratistatin; Narciclasine; Ungeremine	Anticancer and antiproliferative in vitro.	Seabrooks & Hu^17^
			Pettit et al.^139^
*Gryllodes sigillatus*	Peptides IIAPPER, KVEGDLK, LAPSTIK, VAPEEHPV, YKPRP, PHGAP and VGPPQ	α-Amylase, a-glucosidase, DPP-IV, ACE inhibition, lipase inhibition and anti-inflammatory.	Hall et al.^140^; Zielinska et al.^130,131^
*Schistocerca gregaria*	Desmosterol, (3β, 5α) cholesta-8, 14, 24-trien-3-ol, 4, 4-dimethyl, (3β, 20 R) cholesta-5, 24-dien-3, 20-diol	Antimicrobial	Cheseto et al.^55^
	α-Glucosidase (AIGVGAIER, GKDAVIV, FDPFPK and YETGNGIK)	ACE and lipase inhibition, antioxidant and anti-inflammatory.	Zielinska et al.^130,131^

Anti-cancer and tumor suppressive effects have been observed for bioactive metabolites such as actinomycin-D, isocoumarins periplatins A-D, (R)-(+)-palasonin, palasonimide, cantharimide, palasonin, cantharidin, norcantharidin, pederin, pancratistatin, narciclasin and ungeremin found in insect species including *Macrotermes natalensis*^22^, *Periplaneta americana*^23^, *Hycleus oculatus*^24^, *Hycleus lunata*^25^, *Paederus sp*^26^ and *Brachystola magna*^17^ by inducing apoptosis and inhibiting cancer cell growth, in addition to inhibiting tumor metastasis and affecting cancer cell energy metabolism.

Hypertension is one of the main risk factors for cardiovascular disease, affecting millions of people every year. Angiotensin-converting enzyme (ACE) plays a key role in the regulation of blood pressure, and its efficacy in the treatment of hypertension has been proven^27^. Protein hydrolysates from insect species belonging to the orders Coleoptera, Diptera, Hymenoptera, Lepidoptera and Orthoptera have demonstrated ACE inhibitory activity. In other research, specific ACE inhibitory peptides from *Bombyx mori*, *Tenebrio molitor*, *Spodoptera littoralis* and *Oecophylla smaragdina* have been identified^27,28^. It has been reported that protein synthesis can release certain amino acids with important physiological regulatory functions including inhibiting severe hypertension and lowering blood pressure as a result of combination of lysine and methionine, and histidine^29^.

Pessina and collaborators^30^ reported anti-hypertensive effects of defatted *Tenebrio molitor* exhibiting strong ACE inhibitory activity with a dose-devendent reduction of svstolic blood pressure in hypertensive rats. Additionally, biactive metabolites such as macrotermycin A-D, natalenamides A-C, blapsols A-D, molossusamides A-C, photoinduced, melanins and ommochromes, aspongopusamides A-D, polyrhadopamines A-E, troline found in *Macrotermes natalensis*^31,32^, *Blaps japanensis*^17,33^, *Hermetia illucens*^34^, *Polyrhachis dives*^35^ were highlighted to have anti-inflammatory properties through effective inhibition of LOX and COX-2 activity, regulation of expression of inflammation- and immunity-related factors to improve the inflammation pathology. By boosting probiotic production and reducing pro-inflammatory cytokines and plasma lipids, immune response and function in humans, particularly in the gastrointestinal tract, would be linked to insect chitin content.

Chitin is the second most abundant biopolymer found in the exoskeletons of arthropods including insects^36^. It has some potential antinutritional properties, particularly when consumed in large quantities. The antinutritional aspects of chitin includes digestibility issues, inhibition of nutrient absorption and interference with protein digestion. Moreover, Chitin is largely indigestible to humans due to a lack of the enzymes needed to break it down, as its structure is resistant to digestive enzymes such as amylase, protease and lipase^37^. Additionally, chitin can bind to certain nutrients, potentially reducing their bioavailability. Furthermore, due to its rigid, fibrous nature, large quantities of chitin can lead to reduced protein absorption or incomplete digestion by interfering with the digestion of proteins and other macromolecules in the stomach^38^.

Glycosaminoglycan, a polysaccharide found in *Gryllus bimaculatus*, showed a significant anti-inflammatory effect against chronic arthritis in mice by inhibiting C-reactive protein (CRP) through suppression of a number of inflammatory biomarkers in vitro^39^. Furthermore, in combination with indomethacin, glycosaminoglycan was more effective than either agent alone in suppressing paw edema^39^. Furthermore, in rats fed a high-fat diet, glycosaminoglycan reduced CRP levels, abdominal and epididymal fat mass and various serobiochemical parameters (phospholipids, aspartate transaminase (AST), alanine transaminase (ALT), total cholesterol and glucose)^40^. Another study in diabetic mice revealed that glycosaminoglycan supplementation reduced blood glucose and LDL cholesterol levels, and increased the activity of antioxidant enzymes, notably catalase, superoxide dismutase and glutathione peroxidase^41^.These findings indicate that the glycosaminoglycan present in *Gryllus bimaculatus* may help reduce the risk of cardiovascular disease.

The A-D blapsols contained in *Blaps japanensis* demonstrated antioxidant properties^42^. Similarly, higher antioxidant activity was found in peptide^43^, photoinduced, melanins and ommochromes^44^ by analyzing DPPH and hydroxyl radical scavenging activities. Moreover, carminic acid found in *Dactylopius coccus* also exhibited antioxidant activity^17^. Insect hydrolysates and peptide fractions have demonstrated antioxidant properties, by contributing to reduce inflammation and oxidative stress by lowering the level of free radicals present in the body^45,46^. Di Mattia and collaborators^37^ reported that water-soluble extracts found in grasshoppers, silkworms and crickets have an antioxidant capacity around five times greater than that of fresh orange juice in vitro, due to their higher protein/peptide content.

Cantharidin^17^ and defensins-DLP2 and DLP4^47^ found in *Hycleus lunata* exhibited immunomodulatory effects by promoting the expression of immune-related factors, enhancing natural killer cell activity as well as stimulating and activating the innate immune system. Furthermore, protein-enriched fraction from *Musca domestica* also showed immunomodulatory effects^48^. Moreover, immunomodulatory hexapeptide from alcalase hydrolysate of ultramicro-pretreated present in *Bombyx mori* pupae protein has potential therapeutic value as an immunomodulatory bioactive metabolite^49^. Purified polypeptide components (BPP-21 and BPP-22) found in *Apis mellifera* pupae revealed immunomodulatory activity in vivo and in vitro by increasing the phosphorylation of ERK and p38, and modulating the expression of intranuclear transcription factors (EIK-1, MEF-2 and CREB) in the MAPK signaling pathway^50^.

Pessina and collaborators^30^ observed neuroprotective effects in defatted *Tenebrio molitor* larvae. Additionally, bioactive compounds such as Coprismycin A-B and Collismycin A contained in *Paederus sp* have shown neuroprotective effects^26^. Moreover, polybioside^51^ and tetraponerins^52^ present respectively in *Polybia paulista* and *Tetraponera rufonigra* revealed neuroprotective effects.

Anti-microbial effects have been observed in several insect bioactive metabolites, such as Shellolic acid A found in *Kerria lacca*^53^, macrocarpal and grandinol found in *Amauronematus amplus*, *Arge sp*, *Dineura pullior*, *Nematus brevivalvis*, *Nematus pravus*, *Nematus viridescens*, *Nematus viridis*, *Perga affinis*, *Pristiphora alpestris*, *Trichiosoma scalesii*^54^, Desmosterol, (3β, 5α) cholesta-8, 14, 24-trien-3-ol, 4, 4-dimethyl, (3β, 20 R) cholesta-5, 24-dien-3, 20-diol found in *Schistocerca gregaria*^55^.

The antimicrobial effect of *Tenebrio molitor* and *Zophobas morio* has been proven in reducing *E. coli* and *Salmonella* infections in broilers^56^ due to their chitin content, which is a polymer of b-1, 4N-acetylglucosamine and is the primary component of the insect exoskeleton^57,58^. The latter and its degraded products, such as chitosan, exert antimicrobial, antioxidant, anti-inflammatory, anticancer and immunomodulatory activity^59^. Moreover, Nino et al.^60^ and Torres-Castillo et al.^61^ reported potential bioactivity of insect phenolics including tricin, luteolin, apigenin, orientin, iso-orientin, vitexin, iso-vitexin, kaempferol, quercetin, isorhamnetin, myricetin, ferulic acid, sinapic acid, gallic acid, 4-hydroxybenzoic acid, syringic acid, p-coumaric acid, caffeic acid, ferulic acid, sinapic acid, linked to chronic diseases such as antioxidant, anti-inflammatory, and anticancer, among others. Chitooligosaccharides, depolymerized products of chitin and chitosan, taken orally for eight weeks significantly reduced the level of the pro-inflammatory cytokine TNF-α and interleukin (IL)-1β in elderly people^62^.

Antibacterial activity was observed for actinomycin-D, macrotermycin A-D and pseudoxyallemycin-B present in *Macrotermes natalensis*^22,31,63^, 1-(2,5-Dihydroxyphenyl)-3-hydroxybutan-1-one, Roseoflavin and 8-methylamino-8-demethyl-d-riboflavin found in *Odontotermes formosanus*^64,65^, roseoflavin, 8-methylamino-8-demethyl-D-riboflavin, natalamycin and termisoflavones A-C present in *Macrotermes spp*^65,66^, molossusamides A-C found in *Catharsius molossus*^67^, lenzimycins A-B found in *Onthophagus lenzii*^68^, α-pyrone, diketopiperazine, pyrone derivatives, diketopiperazine, photoinduced, melanins and ommochromes found in *Hermetia illucens*^69,70^, and papilistatin found in *Byasa polyeuctes*^71^.

Bioactive metabolites found in insects such as 5-Hydroxyramulosin and biatriosporin-M found in *Odontotermes formosanus*^64^, natalamycin-A, geldanamycin, reblastatin, banegasin, cyclo-NMe-L-3,5-dichlorotyrosine-Dhb and rubrominin A-B found in *Macrotermes natalensis*^22^, efomycin K, efomycin L, efomycin M, efomycin G, elaiophylin, roseoflavin, 8-methylamino-8-demethyl, D-riboflavin, natalamycin, termisoflavones A-C present in *Macrotermes spp*^65,72^, tricin, palmitinic acid and eicosane found in *Holotrichia diomphalia*^73^ have shown antifungal effects.

Insect-based feeding is associated with the production of short-chain fatty acids (SCFAs), in terms of increasing the abundance and diversity of beneficial bacteria in the gut. One study showed that chitin is broken down into propionate and butyrate SCFAs by the gut microbiota^58^, followed by a reduction in blood cholesterol and triglyceride levels in chickens fed insect meal, with an increase in energy^58^. An increase in white blood cells, haemoglobin and red blood cells, followed by improved immune function was observed in fish supplemented with chitin and chitosan^74^. A reduction in triglyceride and cholesterol levels and an increase in blood calcium levels were observed in chickens supplemented with *H. illucens* larvae. This is explained by the fact that chitin’s positive charge enables it to bind negatively charged free fatty acids and bile acids^75^.

There are a variety of hypoglycemic bioactive metabolites in insects and their products, including proteins, peptides, polysaccharides, unsaturated fatty acids, alkaloids, and flavonoids^76^. Silkworm hydrolysate and fibroin are said to be ideal blood sugar regulators^53^. In addition, silkworm larvae, honey and chrysalises contain a large number of polysaccharides with hypoglycemic effects^77^. Removing the acetyl group, chitin is transformed into soluble chitosan. The oligosaccharides obtained by enzymolysis or acid hydrolysis of chitosan also have hypoglycemic effects in humans^78^. Insect fat is rich in unsaturated fatty acids^79^, including linoleic acid, which can improve glucose tolerance, with effects on insulin and reduces the incidence of cardiovascular and retinal complications in diabetic patients^80^.

Studies on trace elements show that magnesium, zinc, calcium, iron, copper, chromium, nickel, selenium among others are linked to human blood sugar metabolism with hypoglycemic effects^81^. Additionally, edible insects contain high levels of linolenic acid, which can prevent the synthesis of fatty acids and glycyrrhizin and accelerate the β-oxidation of fatty acids. Linolenic acid functions to reduce triacylglycerides, prolong clotting time and combat thrombosis, and is widely present in lepidopterous larvae, such as *Clanis bilineata tsingtauica* Mell, *Tenebrio molitor*, *Zophobas atratus*^82^. Moreover, chitin and chitosan present in *Tenebrio molitor* larvae can reduce blood pressure, blood lipids, and promote cholesterol metabolism^78,83^.

Moreover, Teixeira et al. ^84^ reported 177 peptides with predicted bioactivities and 61 peptides with bioactivity assessed In vitro and 3 peptides with bioactivity assessed In vivo from *Gryllodes sigillatu*, *Gryllus assimilis*, *Schistocerca gregaria, Alphitobius diaperinus, Tenebrio molitor,Polyphylla adspersa, Apis mellifera, Oecophylla smaragdina, Bombyx mori, Spodoptera littoralis, Hermetia illucens*, and *Musca domestica*.

## Purification and identification of bioactive metabolites found in edible insects

### The purification of bioactive metabolites from edible insects

Several key techniques and methodologies are being used to isolate, identify and purify bioactive metabolites present in the tissues of edible insects. These bioactive metabolites are of growing interest due to their potential health benefits, including antimicrobial, antioxidant, anti-inflammatory and anticancer properties. The general process for purifying bioactive metabolites from edible insects is described below.

#### Sample preparation and extraction

Proper sample preparation and extraction is the first step in the purification of bioactive metabolites from edible insects. The insect species selected can vary according to the bioactive compounds sought^85^. Depending on the solubility of the target metabolites, bioactive compounds can be extracted using a variety of solvents, including methanol and ethanol for extracting polar compounds like polyphenols and peptides^86^, hexane for lipid-soluble compounds such as fatty acids and sterols^87^, water for hydrophilic bioactive compounds, especially antioxidants^88^, and acetone is also used for both lipid and protein extractions^89^. Once extraction is complete, the resulting solution is usually concentrated using techniques such as rotary evaporation to remove the solvent. In addition, filtration is performed to remove insoluble solids, leaving a clear extract ready for further purification.

#### Purification Techniques

Once extraction is complete, techniques such as rotary evaporation are used to remove the solvent and concentrate the solution. In addition, insoluble solids are removed by filtration, leaving a clear extract ready for further purification. After concentration, bioactive metabolites are purified using chromatographic and separation techniques including High-Performance Liquid Chromatography (HPLC), one of the most common methods for separating and purifying bioactive metabolites from insect extracts^90^, Gas Chromatography (GC) which is particularly particularly useful for purifying volatile compounds, such as fatty acids and terpenoids^91^, Thin-Layer Chromatography (TLC), this one can be used as a preliminary purification step for lipophilic compounds such as sterols and antioxidants, even though not as advanced as HPLC; Size-Exclusion Chromatography (SEC): SEC is beneficial technique for separating compounds based on their molecular size. Very useful when purifying large molecules like proteins or polysaccharides from insect exoskeletons^92^, and Ion-Exchange Chromatography which is a method particularly used for isolating charged compounds, such as bioactive peptides^93^.

#### Characterization of purified bioactive metabolites in edible insects

After purification, isolated metabolites are characterized to confirm their identity as well as their bioactivity using several techniques including mass spectrometry (MS): a powerful tool for identifying the molecular weight and structure of bioactive metabolites^94^, nuclear magnetic resonance (NMR): often used for detailed structural characterization of purified metabolites, particularly to identify complex molecules such as fatty acids and peptides; and UV-Vis spectrophotometry, which is frequently used to identify and quantify light-absorbing bioactive compounds, including polyphenols and flavonoids^95^.

### Identification of bioactive metabolites in edible insects

The identification of bioactive metabolites in edible insects has garnered much attention due their potential health benefits, such as antimicrobial, antioxidant, anti-inflammatory and even anticancer properties, due to their wealth of bioactive compounds, including peptides, lipids, polyphenols, vitamins, minerals and chitin derivatives^96^. Insects are rich in proteins which can be hydrolyzed to release bioactive peptides with potential health-promoting properties including antimicrobial, antihypertensive by inhibiting angiotensin-converting enzyme (ACE), and antioxidant effects^97^.

Moreover, edible insects are caracerized by a variety of lipids, including essential fatty acids important for human health. Insects such as crickets, mealworms and grasshoppers contain polyunsaturated fatty acids (PUFAs), notably omega-3 and omega-6 fatty acids^98^. Furthermore, many edible insects are rich in polyphenolic compounds, particularly phenolic acids and flavonoids, known for their antioxidant in cells and tissues, free radical scavenging activity and potentially anti-cancer properties^61^.

Additionally, edible insects contain essential vitamins and minerals that support various bodily functions including metabolism, immune function, wound healing, bone health, red blood cell production, and maintaining a healthy nervous system^8^. Other bioactive metabolites such as sterols and triterpenoids are found in the lipids of insects and are known to contribute cardiovascular health by lowering cholesterol, reduce inflammation, and exhibit anticancer properties^99^. In addition to chitin, other polysaccharides such as glucans found in the hemolymph of insects have been studied for their potential bioactivity including anticancer and immunomodulatory properties by stimulating the immune system and improving resistance to infections^98^.

## Consumer attitudes toward edible insects

Consumer attitudes toward edible insects have been a subject of interest and debate in recent years^19^. As the world grapples with the challenges of sustainable food production and environmental concerns, edible insects have emerged as a potential solution to address these issues^14^. However, the acceptance and adoption of edible insects as a mainstream food source largely depends on consumer attitudes and perceptions^21^.

One of the primary factors influencing consumer attitudes toward edible insects is cultural and societal norms^100^. In many Western countries, insects are not traditionally part of the culinary landscape and are often associated with disgust or considered as pests^101^. This deeply ingrained cultural bias leads to a significant barrier to acceptance. However, in other cultures, such as parts of Asia, Africa, and Latin America, insects have long been consumed and are even considered delicacies^102^. Cultural exposure and familiarity with edible insects play a crucial role in shaping consumer attitudes and acceptance^103^.

Many people are concerned about the safety of consuming insects, particularly regarding potential allergenic reactions or contamination^104^. However, numerous studies have shown that edible insects are safe for human consumption when sourced from reliable and regulated suppliers^105^. In fact, insects are often rich in protein, vitamins, and minerals, making them a nutritious and sustainable food option^9^. Education and awareness campaigns highlighting the nutritional benefits and safety standards associated with edible insects can help reshape consumer attitudes.

Traditional livestock production, such as cattle farming, is resource-intensive and contributes to greenhouse gas emissions and deforestation. In contrast, insects require minimal resources, emit fewer greenhouse gases, and can be reared on organic waste, making them an environmentally friendly alternative^106^. Consumers who are conscious of these environmental issues may be more open to incorporating insects into their diet as a sustainable choice^5,107,108^.

The way edible insects are marketed and presented to consumers can significantly impact their perception and willingness to try them^109^. Manufacturers and retailers should focus on creating appealing and visually appealing products that align with consumers’ taste preferences and dietary habits^110^. Clever marketing strategies that emphasize the novelty, sustainability, and health benefits of edible insects can help overcome initial resistance and spark curiosity among consumers^111^.

Furthermore, taste preferences are often developed through exposure and personal experiences. Offering opportunities for consumers to sample and taste insect-based products in a non-threatening and controlled environment can help overcome the initial resistance and foster positive experiences^110^. Social influences, such as peer recommendations and endorsements from influential figures, can also sway consumer attitudes and drive acceptance. Overcoming cultural biases, addressing safety concerns, and raising awareness about the nutritional and environmental benefits of edible insects are crucial steps in reshaping consumer attitudes. By actively engaging consumers, providing appealing product options, and dispelling misconceptions, edible insects have the potential to become a viable and sustainable food source in the future.

**Fig. 3:**
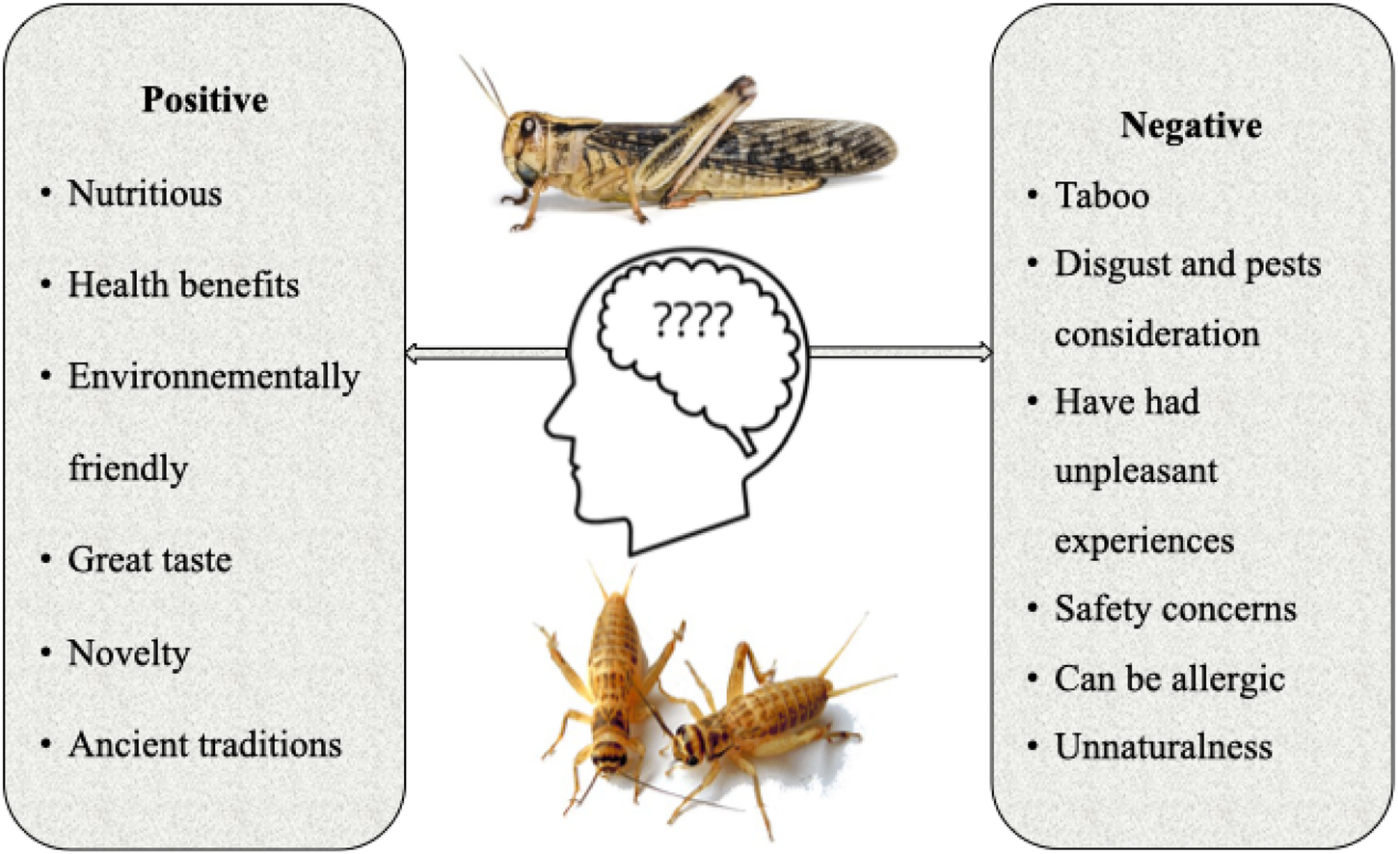
Attitudes towards edible insects as food.

It can be concluded that some of the main positive factors influencing attitudes towards insects include nutritional potential, health benefits, environmentally friendly, great taste, and traditions; on the other hand, the main factors underlining negative attitudes towards insects are, among others, taboo, safety concerns, unpleasant past experiences, allergies and unnaturalness as summarized in Fig. 3.

## Conclusion and future perspectives

It can be generally concluded that insect bioactive metabolites, including marcocarpal, grandinol, trolline, pancratistatin, narciclasin, ungeremin, cantharidin, cordycepin, roseoflavin, lecithin, reblastatin, chitin, chitosan and desmosterol play a crucial role in conferring several beneficial biological activities, such as tumor suppression, anticancer, antihypertensive, anti-inflammatory, antioxidant, immunomodulator, neuroprotective, glycemic and lipid regulation, blood pressure reduction, regulation of intestinal bacterial flora and cardiovascular protection among others. However, proper sample preparation and extraction is the first step in the purification of bioactive metabolites from edible insects. After concentration, bioactive metabolites are purified using chromatographic and separation techniques including High-Performance Liquid Chromatography (HPLC), Gas Chromatography (GC), Thin-Layer Chromatography (TLC), Size-Exclusion Chromatography (SEC). It is noteworthy that nutritional potential, health benefits, environmentally friendly, great taste, traditions, taboo, safety concerns, unpleasant past experiences, allergies, and unnaturalness are among the main factors influencing attitudes towards insects.

Given the immense insect biodiversity, more in-depth investigations should focus on undiscovered bioactive metabolites, for more information on their potential as a sustainable therapeutic source. Particular attention should be paid to increasingly describing the therapeutic benefits and modes of action of insect bioactive metabolites. Additionally, as many human experiments as possible to explore the biological activities of these bioactive metabolites should also be carried out. Studies focusing on cross-reactivity of edible insects, as well as novelty, smart marketing, and good education can further influence attitudes towards insect consumption.

## Data Availability

The datasets generated or analyzed in the current study are available from the corresponding author upon reasonable request.
